# Mobilization of healthy donors with plerixafor affects the cellular composition of T-cell receptor (TCR)-αβ/CD19-depleted haploidentical stem cell grafts

**DOI:** 10.1186/s12967-014-0240-z

**Published:** 2014-09-02

**Authors:** Sergio Rutella, Perla Filippini, Valentina Bertaina, Giuseppina Li Pira, Lidia Altomare, Stefano Ceccarelli, Letizia P Brescia, Barbarella Lucarelli, Elia Girolami, Gianpiero Conflitti, Maria Giuseppina Cefalo, Alice Bertaina, Tiziana Corsetti, Lorenzo Moretta, Franco Locatelli

**Affiliations:** Department of Pediatric Hematology/Oncology and Transfusion Medicine, IRCCS Bambino Gesù Children’s Hospital, Rome, Italy; Pharmacy Service, IRCCS Bambino Gesù Children’s Hospital, Rome, Italy; Istituto di Ricovero e Cura a Carattere Scientifico Giannina Gaslini, Genoa, Italy; Department of Pediatric Science, University of Pavia, Pavia, Italy; Current address: Division of Translational Medicine, Research Branch, Sidra Medical & Research Centre, Doha, Qatar

## Abstract

**Background:**

HLA-haploidentical hematopoietic stem cell transplantation (HSCT) is suitable for patients lacking related or unrelated HLA-matched donors. Herein, we investigated whether plerixafor (MZ), as an adjunct to G-CSF, facilitated the collection of mega-doses of hematopoietic stem cells (HSC) for TCR-αβ/CD19-depleted haploidentical HSCT, and how this agent affects the cellular graft composition.

**Methods:**

Ninety healthy donors were evaluated. Single-dose MZ was given to 30 ‘poor mobilizers’ (PM) failing to attain ≥40 CD34^+^ HSCs/μL after 4 daily G-CSF doses and/or with predicted apheresis yields ≤12.0x10^6^ CD34^+^ cells/kg recipient’s body weight.

**Results:**

MZ significantly increased CD34^+^ counts in PM. Naïve/memory T and B cells, as well as natural killer (NK) cells, myeloid/plasmacytoid dendritic cells (DCs), were unchanged compared with baseline. MZ did not further promote the G-CSF-induced mobilization of CD16^+^ monocytes and the down-regulation of IFN-γ production by T cells. HSC grafts harvested after G-CSF + MZ were enriched in myeloid and plasmacytoid DCs, but contained low numbers of pro-inflammatory 6-sulfo-LacNAc^+^ (Slan)-DCs. Finally, children transplanted with G-CSF + MZ-mobilized grafts received greater numbers of monocytes, myeloid and plasmacytoid DCs, but lower numbers of NK cells, NK-like T cells and Slan-DCs.

**Conclusions:**

MZ facilitates the collection of mega-doses of CD34^+^ HSCs for haploidentical HSCT, while affecting graft composition.

**Electronic supplementary material:**

The online version of this article (doi:10.1186/s12967-014-0240-z) contains supplementary material, which is available to authorized users.

## Background

HLA-haploidentical hematopoietic stem cell transplantation (HSCT) is an effective therapeutic option for patients with high-risk leukemia, and without human leukocyte antigen (HLA)-matched donors [1]. Historically, clinical success, i.e., full donor-type engraftment in 95% of patients with acute leukemia and negligible incidence of acute and chronic graft-versus-host disease (GVHD), has been achieved with T-cell depleted (TCD) grafts containing a mega-dose of positively selected CD34^+^ cells, without the use of any post-transplant immunosuppression [2].

Granulocyte colony-stimulating factor (G-CSF) is widely employed as mobilizing agent in healthy donors and cancer patients. However, G-CSF-based regimens are associated with a 5-30% failure rate [3]. The bicyclam AMD3100, also known as plerixafor, was approved in 2008 for use in combination with G-CSF to mobilize hematopoietic stem cells (HSC) for autologous HSCT [4]. Plerixafor (Mozobil®, MZ) specifically and reversibly blocks the binding of C-X-C chemokine receptor 4 (CXCR4) to its natural ligand, stromal cell-derived factor 1 (SDF1), a CXC chemokine and key regulator of HSC homing and retention in the bone marrow (BM). We previously showed that G-CSF-mobilized peripheral blood CD34^+^ cells retain surface CXCR4 [5], implying that BM microenvironment might easily accommodate immigrating progenitor cells that express high levels of CXCR4 following G-CSF mobilization or stress conditions. MZ synergizes with G-CSF through its different mechanism of action, as suggested by randomized phase III studies, where plerixafor and G-CSF were shown to be superior to G-CSF alone for CD34^+^ HSC mobilization and collection [6,7].

Dendritic cells (DCs) are professional antigen-presenting cells triggering primary adaptive immune responses through the activation of *naïve* CD4^+^ and CD8^+^ T cells [8]. Initially, human DCs were categorized into type 1 (DC1) and type 2 DCs (DC2), which are functionally distinguished by pattern of cytokine production and T-cell driving capacity. Recently, 3 cell types assigned to the DC lineage have been characterized in human blood, i.e., type 1 myeloid DCs (MDC1), type 2 myeloid DCs (MDC2) and plasmacytoid DCs [9-11]. Blood CD1c^+^ MDC1 efficiently cross-present soluble antigens and prime cytotoxic T cells [12]. Human BDCA-3^+^ MDC2 share some characteristics with murine CD8α^+^ DCs, such as production of high amounts of IL-12p70 and interferon (IFN)-λ [10,11]. By contrast, human plasmacytoid DCs secrete IFN-α and activate natural killer (NK) cells, macrophages and myeloid DCs to mount immune responses against microbial products.

There is growing evidence that the biological activities of G-CSF are not limited only to the myeloid lineage, but extend to other cell types mediating, amongst the others, inflammation, immunity and angiogenesis [13,14]. Initial studies in mice supported a role for G-CSF in immune skewing towards T helper type 2 (Th2) cytokine production [15]. In humans, G-CSF increases IL-4 release and decreases IFN-γ secretion [16], and promotes the differentiation of transforming growth factor-β1/IL-10-producing type 1 regulatory T cells (Treg), which are endowed with the ability to suppress T-cell proliferation in a cytokine-dependent manner [17,18]. Finally, G-CSF indirectly modulates DC function, by inducing hepatocyte growth factor, IL-10 and IFN-α, and mobilizes DC2 [19-21].

Currently, the use of MZ in healthy donors is off-label, with anecdotal reports describing its ‘just-in-time’ application either as single agent or after mobilization failure with G-CSF [22-24]. The few available data on immunological effects of MZ are mostly limited to cancer patients and show that CD8^+^ T-cell release of IFN-γ and TNF-α may be higher in autologous grafts collected after G-CSF and MZ, compared with G-CSF alone [25].

We recently developed a novel graft manipulation strategy aimed at extensively removing T-cell receptor (TCR)-αβ^+^ T cells and CD19^+^ B cells from haploidentical HSCs, prior to their infusion into children with non-malignant disorders [26]. TCR-αβ and B-cell depletion is intended to prevent GVHD and post-transplantation lymphoproliferative disorders, respectively. The present study was designed and conducted to investigate whether and to what extent the administration of MZ, an ‘immediate salvage’ strategy in donors with suboptimal CD34-cell counts after standard-dose G-CSF, affects the cellular composition of the graft in the setting of TCR-αβ/CD19-depleted haploidentical HSCT for children with hematological disorders.

## Methods

### Donor eligibility and treatment plan

**Figure 1:**
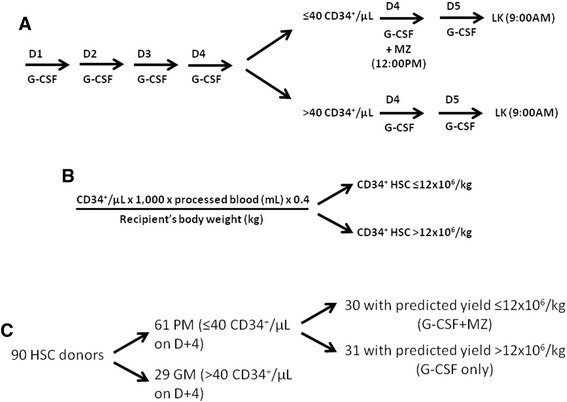
**CD34** ^**+**^ **HSC mobilization algorithm.** G-CSF was given subcutaneously at 12 μg/kg of body weight (divided into 2 doses, 12 hours apart) for 4 consecutive days (D). On day +4, circulating HSCs were enumerated by flow cytometry, as detailed in Materials and Methods. Panels **A** and **B**: If donors had ≤40 CD34^+^ cells/μL of blood on day +4 (and/or a predicted apheresis yield ≤12x10^6^ CD34^+^ HSCs/kg of recipient’s body weight) [27], a single dose of plerixafor (MZ) was administered subcutaneously (0.24 mg/kg of body weight), followed by HSC collection on day +5. If donors had >40 CD34^+^ cells/μL of blood on day +4 (and/or a predicted apheresis yield >12x10^6^ CD34^+^ HSCs/kg of recipient’s body weight), mobilization was continued with G-CSF alone and HSCs were collected on day +5 **(panel C)**.

Ninety healthy HLA-haploidentical parents of children with hematological disorders were enrolled in the present study. Our treatment algorithm is detailed in Figure 1. Donors received MZ on a compassionate basis, outside the approved label of the drug, after providing written informed consent. The study was reviewed and approved by the Institutional Ethics Committee (protocol #938-LB). We opted for an ‘immediate salvage’ strategy administering 0.24 mg/kg MZ to donors failing to achieve the predefined cutoff of ≥40 CD34^+^ HSCs/μL of blood after 4 daily split-doses of 12 μg/kg G-CSF (Figure 1A) and/or donors with suboptimal predicted apheresis yields on the expected day of HSC collection (≤12x10^6^ CD34^+^ HSCs/kg of recipient’s body weight; Figure 1B) [27]. MZ was administered at 12:00 PM (day +4) and leukapheresis was performed on day +5, following the morning dose of G-CSF (i.e., 9 hours after MZ injection). Peripheral blood was collected prior to G-CSF (day 0) and at peak CD34^+^-cell counts (days +4 and +5). Normal BM samples were collected from 10 consented donors performing BM donation under general anesthesia for matched-sibling HSCT.

### Antibodies and reagents

FITC-conjugated anti-IFN-γ, PE-conjugated anti-IL-17A, PerCP-Cy5.5-conjugated anti-CD4 and APC-conjugated anti-IL-4 monoclonal antibodies (mAbs; Human Th1/Th2/Th17 Phenotyping Kit), Lineage Cocktail-1 (a mixture of FITC-conjugated mAbs directed against CD3, CD14, CD16, CD19, CD20 and CD56), BD™ Stem Cell Enumeration Kit™, mAbs directed against human CD14, CD16, CD45RO, CD62L, CD19, IgD, CD27 and CD184 (12G5 clone), Cytofix/Cytoperm™ solution and Golgi Plug Protein Transport Inhibitor™ were purchased from BD Biosciences (Mountain View, CA). 6-sulfo-LacNAc^+^ (Slan)-DCs were identified using a PE-conjugated anti-M-DC8 mAb (DD-1 clone; Miltenyi Biotec, Bologna, Italy). Fluorochrome-conjugated mAbs directed against BDCA-1 (CD1c; AD5-8E7 clone; type 1 myeloid DCs or MDC1), BDCA-2 (CD303; AC144 clone; plasmacytoid DCs), BDCA-3 (CD141 or thrombomodulin; AD5-14H12 clone; type 2 myeloid DCs or MDC2) and BDCA-4/neuropilin-1 (CD304; AD5-17 F6 clone; plasmacytoid DCs) were from Miltenyi Biotec. Phorbol myristate acetate (PMA) and ionomycin were purchased from Sigma-Aldrich (Milan, Italy). The mAb panel and mAb combinations used for graft characterization are detailed in Table 1.

**Table 1:** **Antibody panel used for graft characterization**

**FITC**	**PE**	**Per-CP**	**APC**	**Events analyzed (#)**
CD20	-	CD45	CD3	500,000
TCR-γδ	TCR-αβ	CD45	CD3	500,000
CD45	CD34	7-AAD	-	>100 CD34^+^ events
CD14	Slan-DC	-	CD16	50,000
CD3	CD16+CD56	CD45	CD19	50,000
CD8	CD4	CD45	CD3	50,000
Lineage 1	BDCA-4	-	BDCA-2	50,000
Lineage 1	BDCA-3	-	BDCA-1	50,000

FITC = fluorescein isothiocyanate; PE = phycoerythrin; Per-CP = Peridinin-Chlorophyll-Protein; APC = allophycocyanin; TCR = T-cell receptor; 7-AAD = 7-amino-actinomycin-D; BDCA = blood dendritic cell antigen.

### Enumeration of CD34^+^ cells

CD34^+^ HSCs in donor PB, leukapheresis products and manipulated grafts were counted using the ISHAGE protocol [28].

### TCR-αβ/CD19 immunomagnetic depletion

Leukapheresis collections containing <60.0x10^9^ nucleated cells were washed by centrifugation at 300 *g* for 15 minutes with PBS-EDTA-0.5% human serum albumin (Clini-MACS® washing buffer; Miltenyi Biotec) and were treated with γ-globulins to minimize the non-specific antibody binding to Fc receptors, before the addition of the biotin-conjugated, anti-TCR-αβ antibody. Cells were then incubated with magnetic beads conjugated to anti-biotin and to anti-CD19 antibodies, were re-suspended at <300.0x10^6^/mL and were applied to the fully automated Clini-MACS® device [29].

### Enumeration of B-cell and T-cell subsets

The following B-cell subsets were monitored in HSC donors: *naïve* B cells (CD19^+^CD27^−^IgD^+^), switched memory B cells (CD19^+^CD27^+^IgD^−^), non-switched memory B cells (CD19^+^CD27^+^IgD^+^) and double-negative memory B cells (CD19^+^CD27^−^IgD^−^). [30] Based on CD45RO and CD62L expression, T cells were allotted to either of the following subpopulations: *naïve* T cells (T_N_; CD45RO^−^CD62L^+^), effector memory T cells (T_EM_; CD45RO^+^CD62L^−^), central memory T cells (T_CM_; CD45RO^+^CD62L^+^) and terminally differentiated effector T cells (T_EFF_; CD45RO^−^CD62L^−^) [31].

### Enumeration of NK cells

Three NK-cell subsets were identified and counted. Based on their reciprocal expression of CD16 and CD56, CD3^−^ cells within the lymphoid gate were subdivided into fully mature NK cells (CD56^+^CD16^+^), tissue-resident NK cells (CD56^+^CD16^−^) and immature NK cells (CD56^−^CD16^+^) [32].

### Enumeration of monocyte and DC subsets

Three monocyte subsets were analyzed based on CD14 and CD16 expression: classical (CD14^++^CD16^−^), intermediate (CD14^++^CD16^+^) and non-classical monocytes (CD14^+^CD16^++^) [9]. Intermediate and non-classical monocytes were collectively referred to as CD16^+^ monocytes. To monitor DC mobilization, cells were stained with the ‘Lineage Cocktail-1’ and with mAbs directed against CD1c, CD303, CD141, or CD304 [33]. After gating on Lineage^−^ events, DCs were enumerated and their frequency was expressed as a percentage of total leukocytes [9,33]. 6-sulfo-LacNAc^+^ (Slan)-DCs were counted with the anti-M-DC8 antibody recognizing an O-linked carbohydrate modification of P-selectin glycoprotein ligand-1 [34].

### Intracellular cytokine staining

Cytokine production at the single-cell level was assessed with mAbs directed against IFN-γ, IL-17 and IL-4. CD4^+^ cells were activated for 4–6 hours with 50 ng/mL PMA and 1 μg/mL ionomycin, in the presence of inhibitors of protein transport. Following fixation and permeabilization, cells were labeled with cytokine-specific mAbs for 30 minutes at 4°C and then analyzed by flow cytometry.

### Immunofluorescence analysis

After staining for surface antigens with mAbs at 4°C for 30 minutes, cells were incubated with 0.9% ammonium chloride for 5 minutes to lyse residual red blood cells. Cells were then extensively washed with PBS – 1% BSA and were run on a FACS Canto II® flow cytometer (BD Biosciences) with standard equipment. A minimum of 50,000 events was collected and acquired in list mode using the FACS Diva® software package (BD Biosciences).

### Statistical methods

The approximation of data distribution to normality was tested preliminarily using statistics for kurtosis and symmetry. Data were presented as median and range, and comparisons were performed with the Mann–Whitney *U* test for paired or unpaired data, or with the Kruskal-Wallis test with Bonferroni’s correction for multiple comparisons, as appropriate. *P* values ≤ 0.05 denoted statistical significance.

## Results

### MZ in adjunct to G-CSF potently mobilizes CD34^+^ HSCs in healthy donors

**Figure 2:**
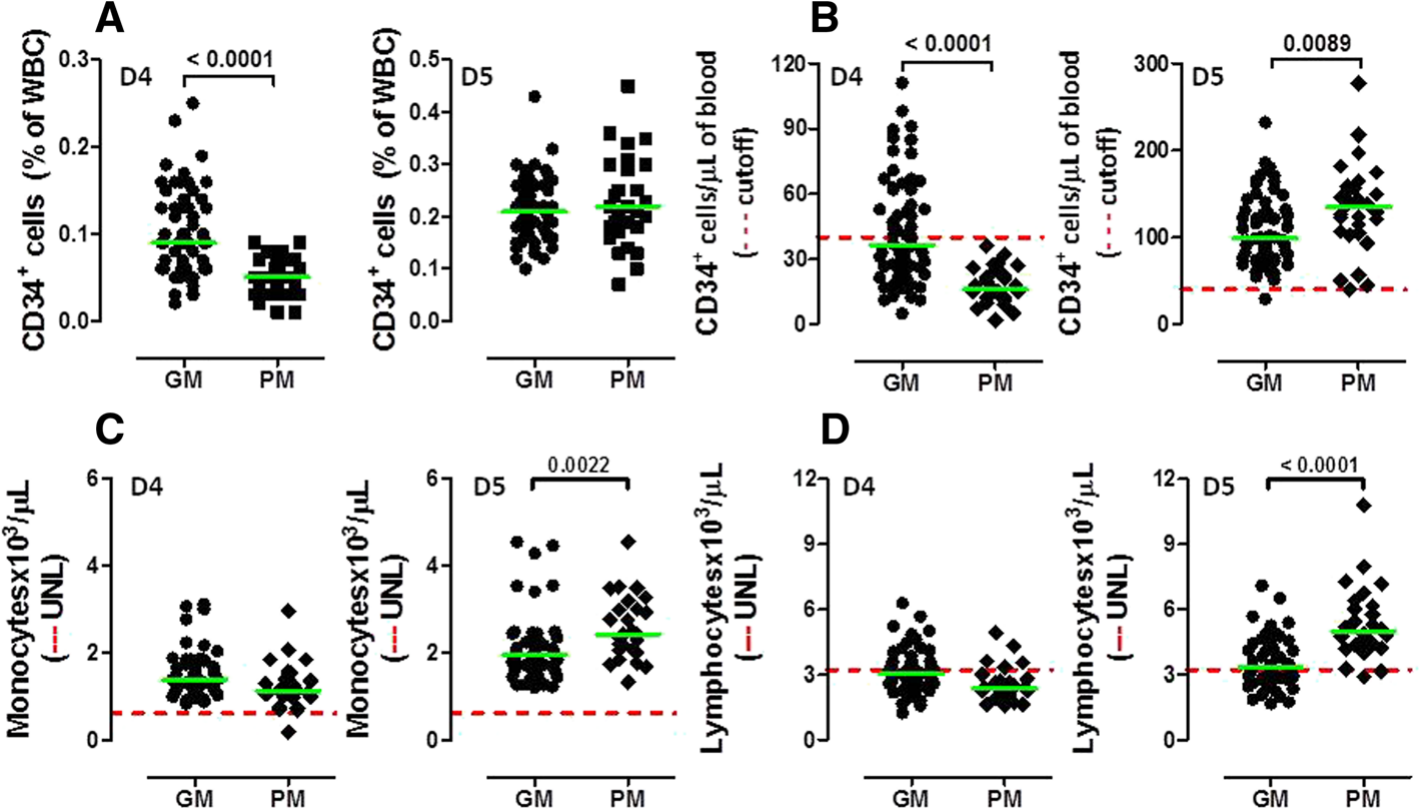
**CD34** ^**+**^ **HSC, monocyte and lymphocyte mobilization with G-CSF and plerixafor (MZ).** Relative frequency **(panel A)** and absolute number **(panel B)** of CD34^+^ HSC, as well as absolute monocyte **(panel C)** and lymphocyte counts **(panel D)**, are shown in ‘good mobilizers’ (GMs) and ‘poor mobilizers’ (PMs) on day +4 (left graph) and day +5 (right graph). The green bar denotes the median value. Comparisons were performed with the Mann–Whitney *U* test for paired data. The dotted red line in panel **B** refers to the predefined cutoff of 40 CD34^+^ HSCs/μL of blood used on day +4 to discriminate the PMs from the GMs. The dotted red line in panels **C** and **D** indicates the upper normal limit (UNL) of monocyte and lymphocyte count. WBC = white blood cells.

We initially enumerated circulating CD34^+^ cells in healthy donors receiving G-CSF for 4 consecutive days. Overall, 61 out of 90 donors (68%) failed to achieve the predefined threshold of 40 CD34^+^ HSCs/μL of blood and were operationally defined as ‘poor mobilizers’ (PM; Figure 1C and Figure 2). However, 31 out of these 61 donors (51%) were re-assigned to the ‘good mobilizer’ (GM) group, since the prediction algorithm favored an apheresis yield >12x10^6^ CD34^+^ HSCs/kg of recipient’s body weight (Figure 1C). By contrast, 29 out of the 90 donors (32%) had post-mobilization CD34-cell counts greater than the cutoff value of 40 CD34^+^ HSCs/μL on day +4, and were classified as ‘good mobilizers’ (GM). Collectively, 60 donors fell into the GM category, whereas the remaining 30 PMs (33% of the whole donor cohort) received single-dose MZ.

In GMs and PMs, the frequency (Figure 2A) and number (Figure 2B) of circulating CD34^+^ HSCs were 0.10% (range 0.02-0.25) and 0.05% (0.01-0.09; p < 0.0001), and 36.5 cells/μL (5–110) and 16.0 cells/μL (2–36; p < 0.0001), respectively, on day +4 of G-CSF administration. Figure 2B also illustrates that CD34^+^-cell counts further increased on day +5 in GMs given G-CSF alone, when compared with those recorded on day +4. PMs consistently achieved >40 CD34^+^ cells/μL of blood after single-dose MZ, and their CD34^+^ counts on day +5 were even higher when compared with those measured in GMs given G-CSF alone [135.0 cells/μL (40–277) vs. 99.0 cells/μL (29–232); p = 0.0089]. Interestingly, PMs given single-dose MZ had higher day-5 monocyte and lymphocyte counts compared with GMs receiving G-CSF alone (Figure 2C-D). The role of MZ in mobilizing monocytes and lymphocytes was further reinforced by the observation that PMs and GMs had similar monocyte and lymphocyte counts on day +4, before receiving single-dose MZ (Figure 2C-D).

As expected, larger numbers of CD34^+^ HSCs were collected, with one single apheresis session, after mobilization with G-CSF + MZ compared with G-CSF alone (Table 2). However, children whose HSC donors were given MZ had a significantly greater body weight compared with children transplanted with G-CSF-mobilized HSC products [45.0 kg (18–74) vs. 20.5 kg (4–66); p < 0.0001].

**Table 2:** **Number of CD34** ^**+**^ **HSCs harvested and infused according to the mobilization regimen**

	**CD34** ^**+**^ **HSC yield**		
	**G-CSF alone**	**G-CSF + MZ**	***P*** **value**
Harvested CD34^+^ HSCs			
*Median*	462.6x10^6^	655.9x10^6^	**0.0008**
*Range*	93.2-1,216	176.5-1,284	
Infused CD34^+^ HSCs			
*Median*	19.83x10^6^/kg	16.83x10^6^/kg	**0.0044**
*Range*	7.5-79.0	5.8-29.21	

MZ = plerixafor (Mozobil^®^); HSC = hematopoietic stem cells. Comparisons between groups were performed with the Mann-Whitney *U* test for unpaired determinations.

### Effects of MZ on circulating immune cells

**Figure 3:**
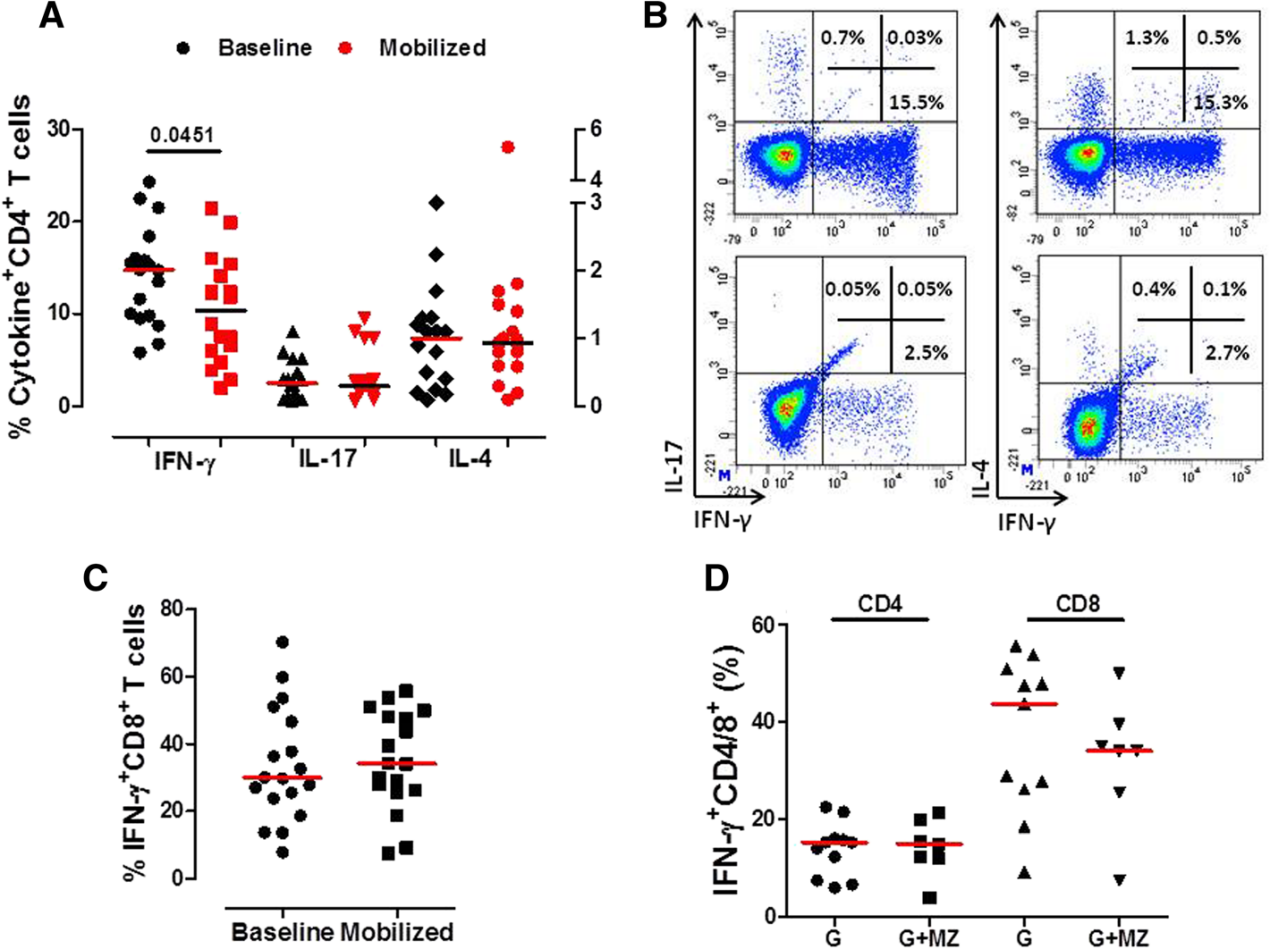
**T-cell cytokine secretion profile after mobilization with G-CSF and plerixafor (MZ).** PBMCs from 18 randomly selected donors given G-CSF alone (G; n = 11) or G-CSF and plerixafor (MZ; n = 7) were stimulated with polyclonal activators *in vitro* and then fixed, permeabilized and stained with anti-IFN-γ/IL-17/IL-4 mAbs. Panel **A**: Percentage of cytokine-expressing CD4^+^ T cells at baseline and after mobilization. Bars denote the median value. Comparisons were performed with the Mann–Whitney *U* test for paired determinations. Panel **B**: A representative experiment for the enumeration of Th1, Th17 and Th2 CD4^+^ T cells is shown. The percentage of cells staining positively for each cytokine is indicated. Markers were set according to the proper isotypic control (not shown). Panel **C**: Percentage of IFN-γ-expressing CD8^+^ T cells at baseline and after mobilization. Bars denote the median value. Panel **D**: Percentage of IFN-γ-expressing CD4^+^ and CD8^+^ T cells in healthy donors assigned to the GM (G-CSF alone) and to the PM group (G-CSF + MZ). Bars denote the median value. Differences were not statistically significant.

It is presently unknown whether single-dose MZ affects T-cell and B-cell phenotype in G-CSF-treated healthy subjects [17,18]. We thus extensively characterized PB samples from donors receiving either G-CSF alone or G-CSF and MZ. No statistically significant differences in the relative proportion of TCR-αβ and TCR-γδ-expressing CD3^+^ T cells were detected, when comparing baseline [94.0% (88.8-98.8) and 5.4% (1.0-9.1), respectively] and post-mobilization samples [95.6% (86.5-99.3) and 4.0% (0.6-12.7), respectively], irrespective of the mobilization protocol (Additional file 1). Moreover, the frequency of T_N_, T_CM_, T_EM_ and terminally differentiated effectors was comparable in baseline and post-mobilization samples. Specifically, T_N_ cells accounted for 29.7% (2.4-62.2) and 30.9% (1.0-58.7), and for 32.2% (2.4-56.4) and 26.6% (2.0-53.4) of the CD4^+^ and CD8^+^ T cells before and after treatment with G-CSF (Additional file 1), indicating that G-CSF administration had no apparent effect on the re-circulation of individual T-cell subsets. We also quantitated B-cell subpopulations in mobilized donors. Again, no appreciable differences were detected in the frequency of *naïve* B cells, double-negative B cells, switched memory B cells and non-switched memory B cells (Additional file 2). Furthermore, the addition of MZ to the G-CSF-based mobilization regimen exerted no measurable effect on the relative frequency of T-cell and B-cell subsets (data not shown). Previous studies showed that G-CSF skews T-cell function towards a regulatory profile, both in mice and in humans [15,17]. We thus measured the frequency of CD4^+^ T cells expressing intracellular IFN-γ, IL-17 and IL-4 at baseline and after mobilization with G-CSF. Figure 3A-B illustrates that the frequency of IFN-γ-expressing CD4^+^ T cells was significantly lower in mobilized PB samples compared with baseline. By contrast, IL-4- and IL-17-producing T cells were unchanged. Intriguingly, no differences were found in the frequency of IFN-γ-expressing CD8^+^ T cells (Figure 3C). When data were dichotomized based on the mobilization regimen used, no statistically significant differences were detected in the frequency of CD4^+^ and CD8^+^ T cells expressing intracellular IFN-γ (Figure 3D).

**Figure 4:**
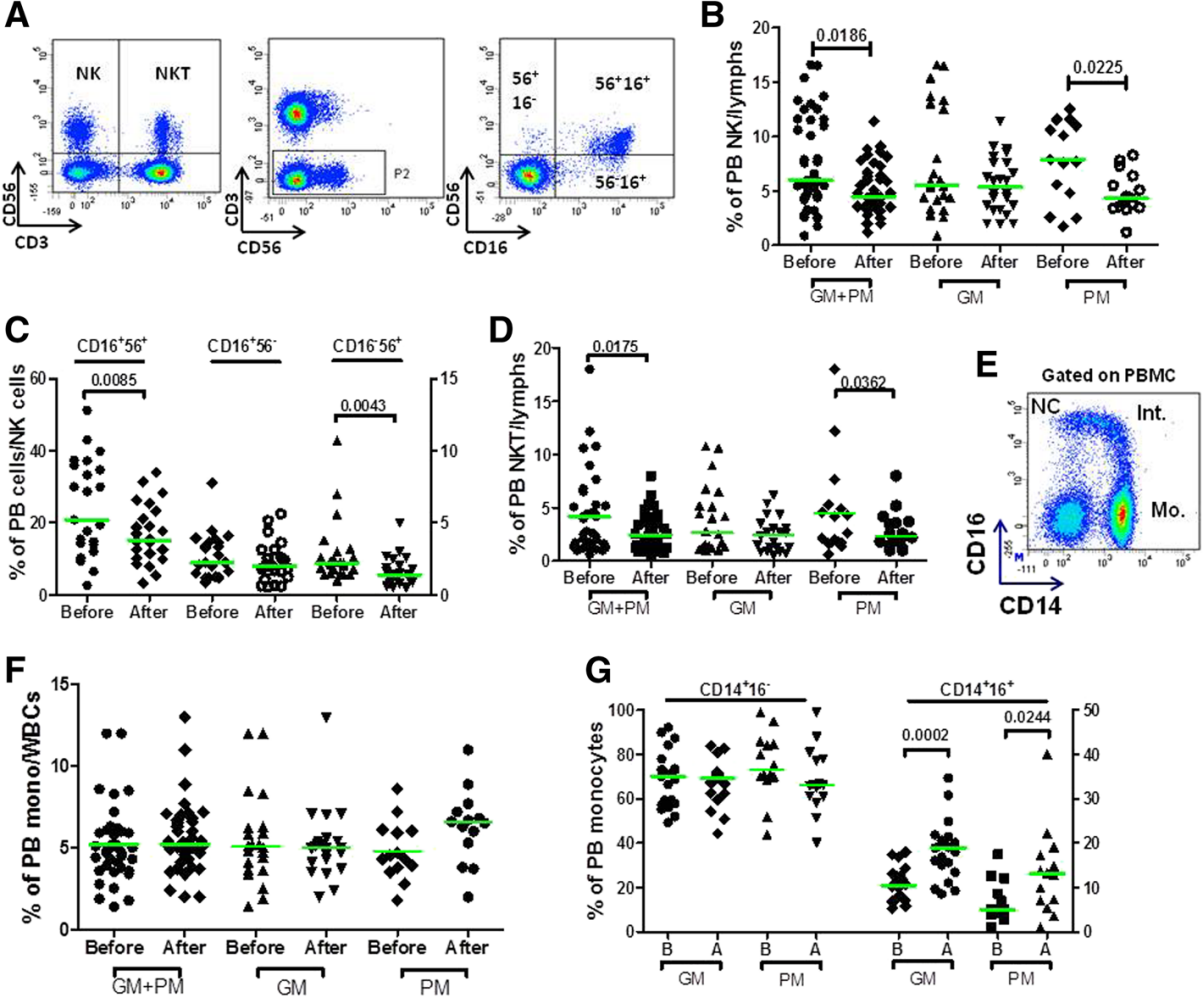
**Mobilization of NK cells, NK-like T cells and monocytes with G-CSF and plerixafor (MZ).** Panel **A**: Donor PBMCs were labeled with mAbs directed against CD3, CD16 and CD56 to enumerate classical NK cells, NK-cell subsets and NK-like T cells. A representative experiment illustrating the gating strategy for the analysis of NK cells and NK-like T cells is shown. After gating on CD3^-^ events (P2), dot plots were used to visualize the reciprocal expression of CD16 and CD56 on NK-cell subsets. Panel **B**: The percentage of NK cells within the lymphoid gate (lymphs) is plotted. PB samples from 40 randomly selected donors were used for this analysis. GM = ‘good mobilizers’; PM = ‘poor mobilizers’; before = baseline samples; after = samples collected after HSC mobilization. Bars indicate the median value. Comparisons were performed with the Mann Whitney *U* test for paired determinations. Panel **C**: The percentage of each subset/total NK cells is plotted on *y* axes. Bars indicate the median value. Comparisons were performed with the Mann Whitney *U* test for paired determinations. Before = baseline samples; after = samples collected after HSC mobilization. Panel **D**: Percentage of NK-like T cells within the lymphoid gate. Bars indicate the median value. Comparisons were performed with the Mann Whitney *U* test for paired determinations. Panel **E**: Strategy to enumerate monocyte subsets in donors’ PB. Cells with a CD14^++^CD16^-^ phenotype were considered to be conventional monocytes (Mo.); conversely, cells with a CD14^+^CD16^+^ (intermediate monocytes, Int.) or a CD14^+^CD16^++^ phenotype (non-conventional monocytes, NC) were referred to as CD16^+^ monocytes [9,55]. No statistically significant differences emerged when comparing the frequency of monocytes before and after G-CSF ± MZ **(panel F)**. WBC = white blood cells. The frequency of CD14^+^CD16^-^ and CD14^+^CD16^+^ monocytes before and after HSC mobilization is depicted in panel **G**. Bars indicate median values. Comparisons were performed with the Mann-Whitney *U* test for paired determinations.

As shown in Figure 4A-B, the overall frequency of PB NK cells was significantly lower after HSC mobilization compared with baseline. This reduction was mainly accounted for by the subgroup of PMs who were given MZ (Figure 4B). Notably, both fully mature CD56^+^CD16^+^ NK cells and tissue-resident CD56^+^CD16^−^ NK cells were significantly lower after G-CSF administration, whereas immature CD56^−^CD16^+^ NK cells were not affected by the HSC mobilization regimen (Figure 4C). Mirroring the above data on NK cells, the frequency of NK-like CD56^+^ T cells was also reduced after G-CSF mobilization, especially in PMs given single-dose MZ (Figure 4D). Collectively, these experiments suggest that NK cells and NK-like T cells may be less represented in healthy donors given G-CSF-based mobilization and that this phenomenon is accentuated by the co-administration of MZ.

**Figure 5:**
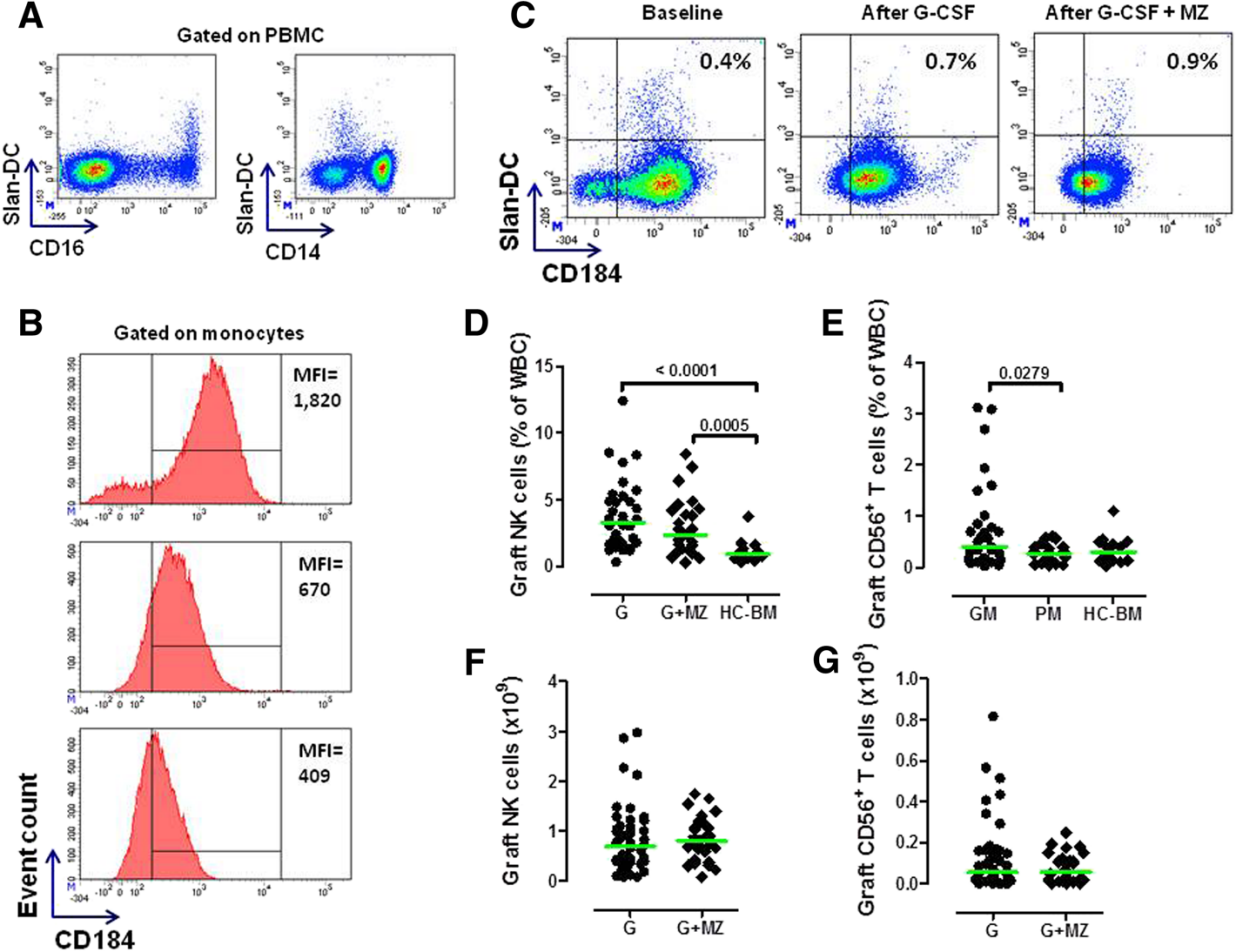
**Expression of CXCR4 (CD184) on Slan-DCs and numbers of NK cells and NK-like T cells in HSC grafts collected after mobilization with G-CSF and plerixafor (MZ).** Panel **A** shows the results of a representative experiment aimed at enumerating 6-sulfo-LacNAc^+^ (Slan)-DCs in donors’ PB. Slan-DCs were consistently CD16^+^ and expressed low-level CD14. Panels **B** and **C**: PB samples from PMs given G-CSF and single-dose MZ were double-stained with anti-CD184 and anti-M-DC8 mAbs to quantitate CXCR4 expression on Slan-DCs. The Mean Fluorescence Intensity (MFI) of CD184 expression on peripheral CD14^+^ monocytes is shown in a representative experiment out of 4 with similar results. The marker was set according to the proper isotypic control. Panel **C** shows preserved levels of CD184 on Slan-DCs after HSC mobilization with G-CSF and MZ. This is in contrast with the marked down-regulation of CD184 on monocytes that is depicted in panel **B**. One representative experiment out of 4 with similar results is shown. Markers were set according to proper isotypic controls. Panels **D**-**G**: Frequency and absolute numbers of NK cells and NK-like T cells in TCR-αβ/CD19-depleted haploidentical grafts. After the immunomagnetic removal of TCR-αβ^+^ T cells and CD19^+^ B cells, mononuclear cells contained within the graft were extensively characterized using mAbs directed against CD3, CD16 and CD56 with the aim at identifying NK cells and NK-like T cells. NK cells and NK-like T cells were also quantitated in aliquots of 10 normal BM samples collected under general anesthesia and intended for HLA-identical sibling HSCT. Comparisons among data sets were performed with the Mann Whitney *U* test for unpaired determinations or with the analysis of variance (ANOVA), as appropriate. HC = healthy control; WBC = white blood cells.

We then aimed at enumerating monocyte and DC subsets in mobilized donors. Classical, intermediate and non-conventional monocytes were labeled and quantitated as shown in Figure 4E. Although the frequency of conventional CD14^+^CD16^−^ monocytes was not significantly different in baseline compared with post-mobilization samples, the proportion of CD16^+^ monocytes increased after G-CSF administration, both in the GM and in the PM group (Figure 4F-G). Slan-DCs (Figure 5A) were not appreciably mobilized into PB by treatment with G-CSF, either alone or combined with MZ (data not shown). Because G-CSF induces CXCR4 cleavage and disrupts the CXCR4/SDF-1α interaction during HSC mobilization, [35] we reasoned that a differential regulation of CXCR4 expression on the surface of Slan-DCs could account for low-level Slan-DC mobilization after combined treatment with G-CSF and MZ. As depicted in Figure 5B, CXCR4 expression significantly decreased on monocytes from donors given G-CSF, after labeling with CD184-12G5 antibodies. Comparable reductions were measured on other leukocyte subsets, such as lymphocytes and neutrophils (data not shown). By contrast, CXCR4 was not apparently down-regulated on circulating Slan-DCs, irrespective of the mobilization regimen (Figure 5C).

Finally, no changes were recorded in the frequency of MDC1, MDC2 or plasmacytoid CD303^+^ or CD304^+^ DCs when comparing baseline and post-mobilization samples, irrespective of the mobilization regimen (Additional file 3) and in keeping with previous reports showing no changes in the frequency of BDCA-2^+^ DCs in donors given G-CSF [36]. Collectively, these experiments suggest that single-dose MZ exerts no major effects on circulating B-cell and T-cell subsets and on monocyte/DC subpopulations, although it may reduce the frequency of blood NK cells and NK-like T cells, when compared with G-CSF alone.

### Effects of MZ on graft composition

In 70 randomly selected HSC donors (43 GMs given G-CSF only and 27 PMs receiving G-CSF plus MZ) undergoing a standard large-volume (15-20 L) leukapheresis, the graft was extensively characterized in terms of immune effector cells with particular relevance to the setting of haploidentical HSCT, such as NK cells, CD3^+^CD56^+^ NK-like T cells, monocytes and DCs. [37-39] For the purpose of comparison, we also enumerated immune effectors in 10 BM samples from healthy HLA-identical sibling donors. TCR-αβ/CD19-depleted grafts obtained after either mobilization regimen were highly enriched with NK cells compared with normal BM samples (Figure 5D). However, the frequency and absolute numbers of NK cells were comparable in grafts collected after the administration of either G-CSF alone or G-CSF and MZ (Figure 5D-E). Although grafts from donors assigned to the GM group had a higher frequency of NK-like CD56^+^ T cells compared with those from the PM group (Figure 5F), the overall number of CD56^+^ NK-like T cells harvested was similar (Figure 5G).

**Figure 6:**
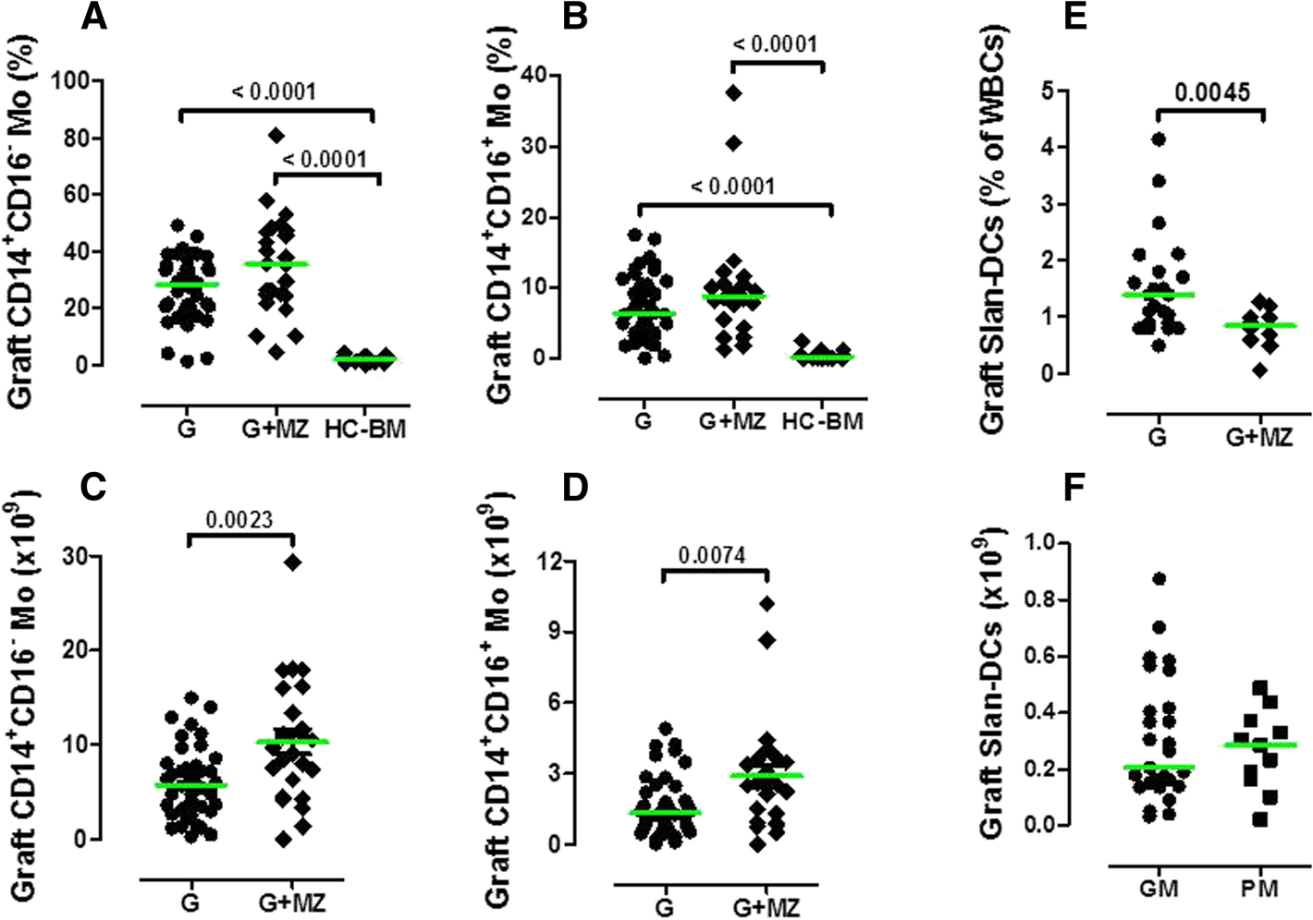
**Numbers of monocytes and Slan-DCs in HSC grafts collected after mobilization with G-CSF and plerixafor (MZ).** After the immunomagnetic removal of TCR-αβ^+^ T cells and CD19^+^ B cells, mononuclear cells contained within the graft were extensively characterized using mAb directed against surface antigens known to be expressed on monocytes and on pro-inflammatory Slan-DCs. Monocytes and Slan-DCs were also quantitated in normal BM samples from matched-sibling donors. The frequency and absolute numbers of CD14^+^CD16^−^ and CD14^+^CD16^+^ monocytes in TCR-αβ/CD19-depleted haploidentical grafts are depicted in panels **A**-**D**. The frequency and absolute number of Slan-DCs are shown in panels **E**-**F**. The green bars indicate median values. GM = ‘good mobilizers’; PM = ‘poor mobilizers’; HC = healthy control. Comparisons among data sets were performed with the Mann Whitney *U* test for unpaired determinations or with the analysis of variance (ANOVA), as appropriate.

Both conventional and CD16^+^ monocytes were contained at higher frequency in TCR-αβ/CD19-depleted grafts, compared with normal BM samples (Figure 6A-B). As shown in Figure 6C-D, both monocyte populations were more abundant in G-CSF + MZ-mobilized grafts compared with grafts collected after G-CSF alone. 6-sulfo-LacNAc^+^ (Slan) DCs constitute 0.5-2% of all PBMCs, are CD14^low^CD16^+^ and are a highly phagocytic monocyte subset inducing potent pro-inflammatory Th1 and Th17 responses [40]. As illustrated by Figure 6E- F, the frequency of Slan-DCs was significantly lower in grafts mobilized with G-CSF + MZ (0.85% of total leukocytes, 0.06-1.3) compared with G-CSF alone (1.4%, 0.5-4.15; p = 0.0045), although this did not translate into the collection of lower numbers of Slan-DCs.

**Figure 7:**
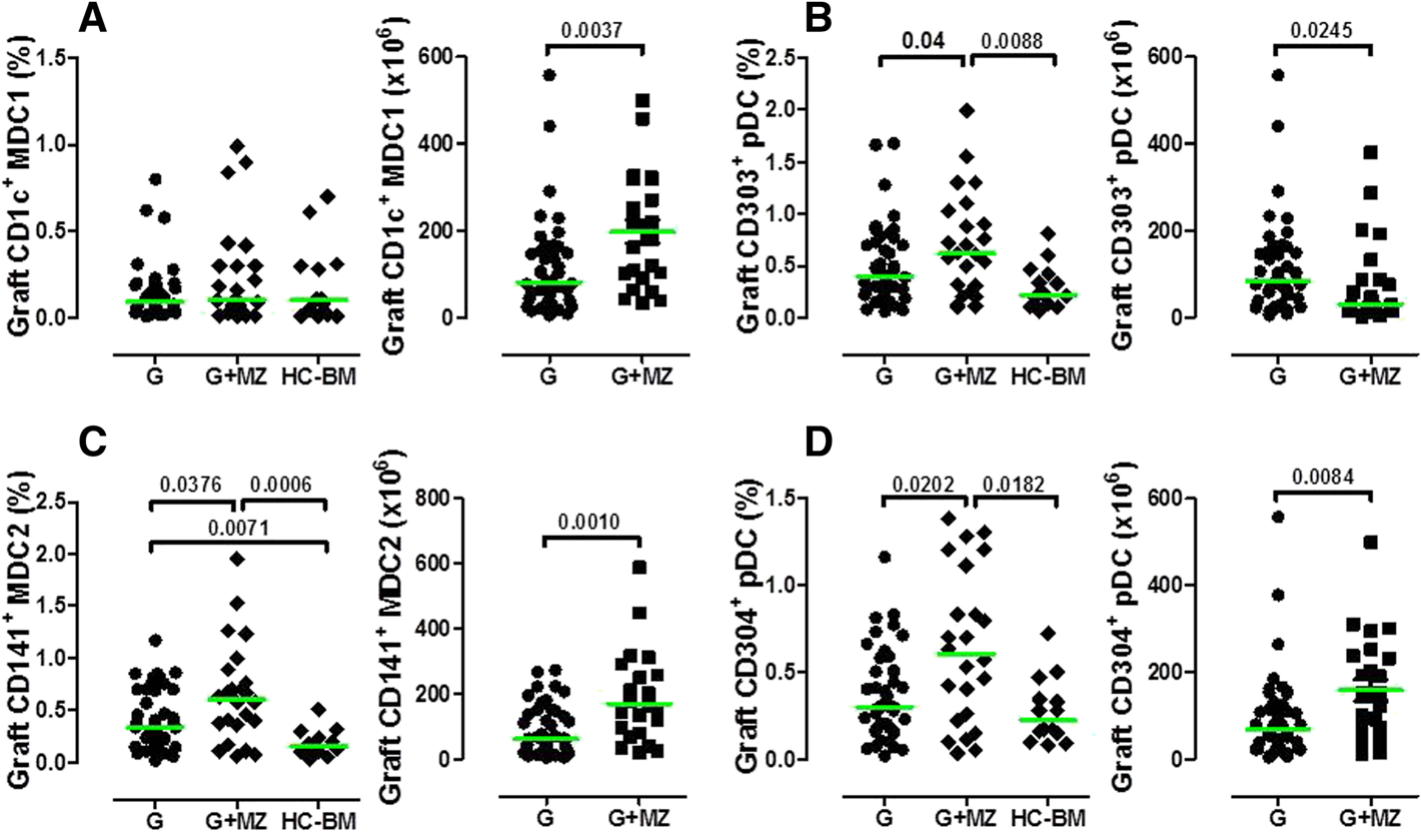
**Numbers of myeloid and plasmacytoid DCs in HSC grafts collected after mobilization with G-CSF and plerixafor (MZ).** After the immunomagnetic removal of TCR-αβ^+^ T cells and CD19^+^ B cells, mononuclear cells contained within the graft were extensively characterized using mAbs directed against surface antigens selectively expressed on DC subpopulations [33]. DC subsets were also quantitated in aliquots of 10 normal BM samples from matched-sibling donors. Panels **A** and **B** depict the frequency and number of MDC1 and plasmacytoid CD303^+^ DCs, respectively, whereas panels **C** and **D** show the frequency and number of MDC2 and CD304^+^ plasmacytoid DCs, respectively. Comparisons among data sets were performed with the Mann Whitney *U* test for unpaired determinations or with the analysis of variance (ANOVA), as appropriate.

Finally, Figure 7 shows that both MDC2 and plasmacytoid DCs were significantly more represented in grafts collected after mobilization with G-CSF and MZ. Thus, the number of MDC1, MDC2 and plasmacytoid DCs collected from donors given G-CSF and MZ was significantly greater. Also, the frequency of DC subsets was higher in TCR-αβ/CD19-depleted grafts compared with normal BM samples (Figure 7). Table 3 summarizes the absolute numbers of cells infused in children receiving the TCR-αβ/CD19-depleted haploidentical grafts collected after G-CSF alone or G-CSF + MZ. Whereas the number of monocytes and DCs infused in the two patient groups was similar, children given G-CSF-mobilized grafts received significantly greater numbers of NK cells and NK-like T cells, as well as pro-inflammatory Slan-DCs, compared with children given HSCT from donors mobilized with G-CSF + MZ (Table 3). These differences can be accounted for by the significantly higher number of nucleated cells collected from GMs treated with G-CSF alone (Table 3). Also, the fact that children receiving haploidentical HSCT from GMs had a lower body weight may have translated into the infusion of higher numbers of NK cells, NKT cells and Slan-DCs.

**Table 3:** **Number of nucleated cells, NK cells, monocytes and DCs infused (per kg of recipient’s body weight) according to the mobilization regimen**

	**G-CSF alone**	**G-CSF + MZ**	***P*** **value**
**Total NC**			
*Median*	1.17x10^9^/kg	0.71x10^9^/kg	**0.0002**
*Range*	0.33-2.99	0.28-1.6	
NK cells			
*Median*	31.3x10^6^/kg	16.8x10^6^/kg	**0.0016**
*Range*	1.8-176.8	2.8-55.9	
NK-like T cells			
*Median*	3.64x10^6^/kg	1.56x10^6^/kg	**0.0016**
*Range*	0.4-34.8	0.2-6.64	
CD16^-^ monocytes			
*Median*	312.2x10^6^/kg	226.8x10^6^/kg	0.34
*Range*	15.9-729.8	28.5-667.0	
CD16^+^ monocytes			
*Median*	74.9x10^6^/kg	63.6x10^6^/kg	0.46
*Range*	1.5-231.7	8.3-169.6	
Slan-DCs			
*Median*	1.40x10^6^/kg	0.85x10^6^/kg	**0.0008**
*Range*	0.5-4.14	0.06-1.27	
CD1c^+^ MDC1			
*Median*	0.84x10^6^/kg	0.88x10^6^/kg	0.88
*Range*	0.1-13.1	0.04-9.66	
CD303^+^ pDCs			
*Median*	4.12x10^6^/kg	4.73x10^6^/kg	0.82
*Range*	0.72-13.66	0.61-22.9	
CD141^+^ MDC2			
*Median*	3.74x10^6^/kg	4.15x10^6^/kg	0.55
*Range*	0.36-14.5	0.33-22.4	
CD304^+^ pDCs			
*Median*	3.8x10^6^/kg	3.1x10^6^/kg	0.78
*Range*	0.2-13.0	0.2-14.7	

NC = nucleated cells; MZ = plerixafor (Mozobil^®^); MDC1 = type 1 myeloid DCs; MDC2 = type 2 myeloid DCs. Comparisons between groups were performed with the Mann-Whitney *U* test for unpaired determinations.

From a clinical standpoint, neither the HSC mobilization regimen (G-CSF alone vs. G-CSF and MZ) nor the number of monocytes and DCs infused correlated with either the occurrence of acute GVHD or the reactivation of viral infections (data not shown). By contrast, patients who developed chronic GVHD had received lower numbers of conventional CD14^+^CD16^−^ monocytes (3.76×10^6^/kg, 1.7-10.1) compared with children without chronic GVHD (11.45×10^6^/kg, 0.6-144.2; p = 0.016].

## Discussion

Herein, we show that the addition of ‘immediate salvage’ MZ to a standard, G-CSF-based mobilization regimen augments HSC yield in HLA-haploidentical donors showing less-than-optimal CD34-cell mobilization, and that MZ administration affects the graft cell composition. Thirty-two percent of our donors were operationally defined as PMs and were given MZ to increase HSC mobilization. More than 95% of the donors collected the required mega-dose of HSCs with a single apheresis session.

Currently, anecdotal reports describe the ‘just-in-time’ application of MZ to healthy donors, either as single agent or after mobilization failure with G-CSF [22-24]. In our donor cohort, single-dose MZ given on day +5 increased the count of CD34^+^ HSCs by 8.2-fold (range 1.4-29.2), compared with that measured after 4 days of G-CSF treatment. This is remarkably similar to the 8-fold increase of CD34^+^ HSCs reported in donors given MZ only [23].

The few available data on immunological effects of MZ are mostly limited to cancer patients and show that CD8^+^ T-cell release of IFN-γ and TNF-α may be higher in autologous grafts collected after G-CSF and MZ, compared with G-CSF alone [25]. We previously showed that G-CSF polarizes human T-cell and DC function towards a tolerogenic profile, implying that G-CSF-mobilized cell therapy products may be intrinsically less capable of inducing uncontrollable GVHD [17,19,41]. This is reinforced by intriguing observations in major histocompatibility complex (MHC)-matched HSCT, where mice given G-CSF-mobilized splenocytes experienced lower rates of skin GVHD compared with recipients of MZ-mobilized splenocytes [42]. In our healthy donors treated with G-CSF, the down-regulation of CD4^+^ T-cell production of IFN-γ was not potentiated by single-dose MZ. Furthermore, IL-17 and IL-4 release by CD4^+^ T cells were not affected by G-CSF and/or MZ administration. These observations are in line with pre-clinical data showing that MZ alone, in contrast to G-CSF, is unable to alter the phenotype and cytokine polarization of T cells, as well as T-cell’s ability to induce acute GVHD [43]. It must be emphasized that, in our study, neither G-CSF nor MZ significantly impaired IFN-γ production by CD8^+^ T cells. Notably, studies in mice suggest that G-CSF may separate GVHD and graft-versus-leukemia (GVL) responses by exerting suppressive effects on CD4^+^ T cells, that are implicated in GVHD, while preserving the cytolytic pathways of CD8^+^ T cells that are critical for effective GVL [44]. At variance with a recent report on the immunological effects of single-dose MZ in healthy donors [42], we were unable to detect any difference in the frequency of naïve B cells after MZ administration. Conceivably, any MZ effect on the recirculation of B-cell subsets may have been obscured by treatment with G-CSF during the 4 days preceding MZ administration. However, neither that study [42] nor our own report identified any modification of CD4 and CD8 T-cell frequencies that could be directly attributable to MZ.

In our cohort of 90 donors, HSC mobilization with G-CSF translated into lowered frequencies of both NK cells and NK-like CD56^+^ T cells, a phenomenon that was mainly accounted for by a reduction of fully mature CD56^+^CD16^+^ and tissue-resident CD56^+^CD16^−^ NK cells, with preserved frequencies of immature CD56^−^CD16^+^ NK cells. Interestingly, the frequency of both NK cells and NK-like CD56^+^ T cells was reduced in the PM group receiving single-dose MZ. Although the number of NK cells collected was not significantly different when comparing donors given G-CSF alone with those receiving G-CSF in combination with MZ, higher numbers of both NK cells and NK-like CD56^+^ T cells were infused in children transplanted with G-CSF-mobilized HSC products. A potential clinical implication of this finding pertains to the field of graft engineering, insofar donor mobilization with G-CSF alone might offer an advantage over the use of G-CSF + MZ for patients with NK-susceptible hematological malignancies [37].

As previously published, G-CSF-mobilized monocytes are functionally defective [45]. In our study, treatment with G-CSF and MZ potently mobilized donor monocytes, especially the CD16^+^ subset of intermediate/non-conventional monocytes. Although the frequency of both conventional and CD16^+^ monocytes was higher in TCR-αβ/CD19-depleted HSC grafts compared with normal BM samples, the overall number of conventional and CD16^+^ monocytes infused in our haploidentical HSCT recipients was not correlated with the mobilization regimen used in the donor. It has been demonstrated that macrophages generated from CD16^+^ monocytes manifest higher phagocytic activity compared with macrophages derived from classical monocytes [46]. In addition, CD14^dim^CD16^+^ monocytes are endowed with a unique patrolling function, as they detect virally infected and damaged cells and produce pro-inflammatory cytokines [47]. In light of these findings, it is conceivable that CD16^+^ monocytes infused with the TCR-αβ/CD19-depleted haploidentical grafts may protect the recipient from infectious episodes, while contributing to prevention of GVHD [48].

There is also evidence that G-CSF mobilizes IL-12/TNF-α-producing, pro-inflammatory Slan-DCs [49]. Thus, Slan-DCs may incite GVHD on the one side, while preserving GVL reactivity on the other side. In our study, the addition of MZ to G-CSF did not affect the mobilization of Slan-DCs. In addition, the frequency of Slan-DCs was lower in HSC grafts collected after the combined treatment with G-CSF and MZ. Because G-CSF administration is associated with *in vivo* cleavage of the N-terminus of CXCR4 on BM-resident HSCs [35], it is tempting to speculate that Slan-DCs, at variance with CD34^+^ HSCs and monocytes [5,50], may not entirely depend upon the CXCR4/SDF-1α axis for re-circulation and homing into lymphoid organs and/or tissues. Our contention is backed by experiments with CD184-12G5 antibodies showing that CXCR4 levels are preserved on the surface of Slan-DCs, but not other leukocyte subsets, analyzed after the *in vivo* administration of G-CSF, when compared to G-CSF plus MZ, this likely favoring Slan-DC retention into tissues. The CD184-12G5 mAbs recognize an epitope involving the first and second extracellular domains of CXCR4, and inhibit MZ binding to CXCR4. A different anti-CXCR4 mAb, termed 1D9, binds to the N terminus of CXCR4 and is not affected by MZ [51]. In a phase 1/2 study of chemo-sensitization with MZ in relapsed or refractory acute myeloid leukemia, a decrease in CD184-12G5 binding was observed from pre-treatment to 6 hours, followed by an increase from 6 to 24 hours, indicating CXCR4 blockade by MZ *in vivo* [52]. By contrast, labeling with CD184-1D9 after MZ treatment revealed an increased expression of CXCR4 between pretreatment and 6 hours, that remained elevated at 24 hours.

Finally, the frequency of CD1c^+^ MDC1, CD141^+^ MDC2 and plasmacytoid DCs [33] was unchanged in PB of donors treated with G-CSF, either alone or in combination with MZ. This is in agreement with previous reports showing no changes in the frequency of BDCA-2^+^ DCs in donors given G-CSF compared with baseline [36]. The TCR-αβ/CD19-depleted haploidentical grafts collected after the administration of G-CSF and MZ were highly enriched with MDC1, MDC2 and plasmacytoid DCs, when compared with normal BM samples. The role played by DCs in the regulation of human GVHD and GVL responses is the subject of intense investigation. Studies of BM transplantation have shown that high numbers of plasmacytoid DCs in the graft correlate with decreased chronic GVHD, at the expense of an increased incidence of leukemia relapse [53]. Conversely, the number of DCs in PB allografts may not predict DC reconstitution kinetics after transplantation or clinical outcome [54]. Importantly, low DC counts at time of engraftment have been associated with worse survival, increased incidence of relapse and higher incidence of grade II-IV acute GVHD [54].

Thus far, we have transplanted 23 children with non-malignant disorders using TCR-αβ/CD19-depleted HSC grafts [26]. Primary graft failure occurred in 4 patients, with 3 patients developing skin-only grade 1 to 2 acute GVHD and no patient suffering from chronic GVHD. The cumulative incidence of transplantation-related mortality was 9.3%. With a median follow-up of 18 months, 21 of 23 children are alive and disease-free, the 2-year probability of disease-free survival being 91.1% [26]. It remains to be determined whether and to what extent the DC content of our TCR-αβ/CD19-depleted HSC grafts and, in particular, the remarkably high numbers of MDC1, MDC2 and plasmacytoid DCs infused correlate with infection control, GVHD and/or leukemia recurrence.

## Conclusions

Collectively, our study shows that MZ is highly effective at mobilizing mega-doses of CD34^+^ HSCs to be transplanted into haploidentical recipients. Furthermore, our data shed some light into the optimal clinical use of MZ, insofar differences in graft cellular composition after mobilization with G-CSF and MZ are expected to quantitatively and/or qualitatively influence the immune processes that occur after allogeneic HSCT.

## Additional files

Additional file 1:
**Phenotype of circulating T-cell subsets after mobilization with G-CSF.** PB samples from 21 randomly selected donors were analyzed for the relative frequency of TCR-αβ/γδ T cells as well as naïve/memory T-cell subsets. Panel A: Lymphoid cells were gated based on their light scatter characteristics and on CD3 expression (P1), followed by the analysis of reciprocal αβ and γδ-chain expression. Panel B: The frequency of CD3^+^ T cells/total lymphoid cells, αβ^+^ T cells/total CD3^+^ T cells and γδ^+^ T cells/total CD3^+^ T cells is shown before and after G-CSF administration. Bars indicate the median value recorded in 21 independent donor samples. Panels C-D: T-cell subsets were identified through labeling with anti-CD62L and anti-CD45RO mAb, which allowed the discrimination of naïve T cells (T_N_) from central-memory T cells (T_CM_), effector-memory T cells (T_EM_) and terminally differentiated effectors. Bars indicate the median value recorded in 21 independent donor samples. TCR = T-cell receptor. Additional file 2:
**Phenotype of circulating B-cell subsets after mobilization with G-CSF.** PB from 21 randomly selected donors were analyzed for the relative frequency of naïve/memory B-cell subsets. Panel A: mAbs directed against CD19, CD27, IgM and IgD were used to discriminate naïve B cells from memory B-cell subsets [30]. Panel B: The frequency of B-cell subsets/total CD19^+^ B cells is shown before and after G-CSF administration. N = *naïve* B cells (CD19^+^CD27^−^IgD^+^); S-M = switched memory B cells (CD19^+^CD27^+^IgD^−^); NS-M = non-switched memory B cells (CD19^+^CD27^+^IgD^+^); DN-M = double-negative memory B cells (CD19^+^CD27^−^IgD^−^). Bars indicate the median value recorded in 21 independent donor samples. Additional file 3:
**Circulating DC subsets after mobilization with G-CSF and plerixafor (MZ).** PB samples from 40 randomly selected donors (24 GMs given G-CSF alone and 16 PMs receiving G-CSF + MZ) were analyzed for the relative frequency of major DC subsets. MDC1, MDC2 and plasmacytoid DCs were identified as detailed in Materials and Methods. Bars denote the median value. Before = baseline samples; after = samples collected after HSC mobilization.
