# Supplementary figures and images for: Mobilization of healthy donors with plerixafor affects the cellular composition of T-cell receptor (TCR)-αβ/CD19-depleted haploidentical stem cell grafts
Source: J Transl Med. 2014 Sep 2;12:240. doi: 10.1186/s12967-014-0240-z (PMC4158047; doi:10.1186/s12967-014-0240-z)

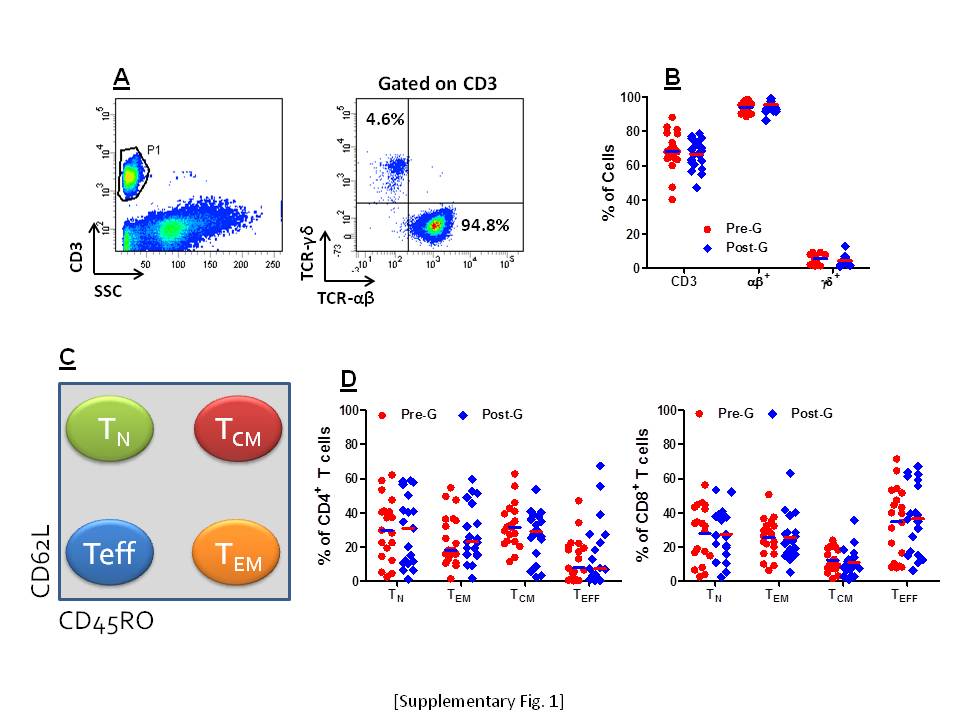

Supplement: Additional file 1: — Phenotype of circulating T-cell subsets after mobilization with G-CSF. PB samples from 21 randomly selected donors were analyzed for the relative frequency of TCR-αβ/γδ T cells as well as naïve/memory T-cell subsets. Panel A: Lymphoid cells were gated based on their light scatter characteristics and on CD3 expression (P1), followed by the analysis of reciprocal αβ and γδ-chain expression. Panel B: The frequency of CD3+ T cells/total lymphoid cells, αβ+ T cells/total CD3+ T cells and γδ+ T cells/total CD3+ T cells is shown before and after G-CSF administration. Bars indicate the median value recorded in 21 independent donor samples. Panels C-D: T-cell subsets were identified through labeling with anti-CD62L and anti-CD45RO mAb, which allowed the discrimination of naïve T cells (TN) from central-memory T cells (TCM), effector-memory T cells (TEM) and terminally differentiated effectors. Bars indicate the median value recorded in 21 independent donor samples. TCR = T-cell receptor. [file 12967_2014_240_MOESM1_ESM.jpeg]

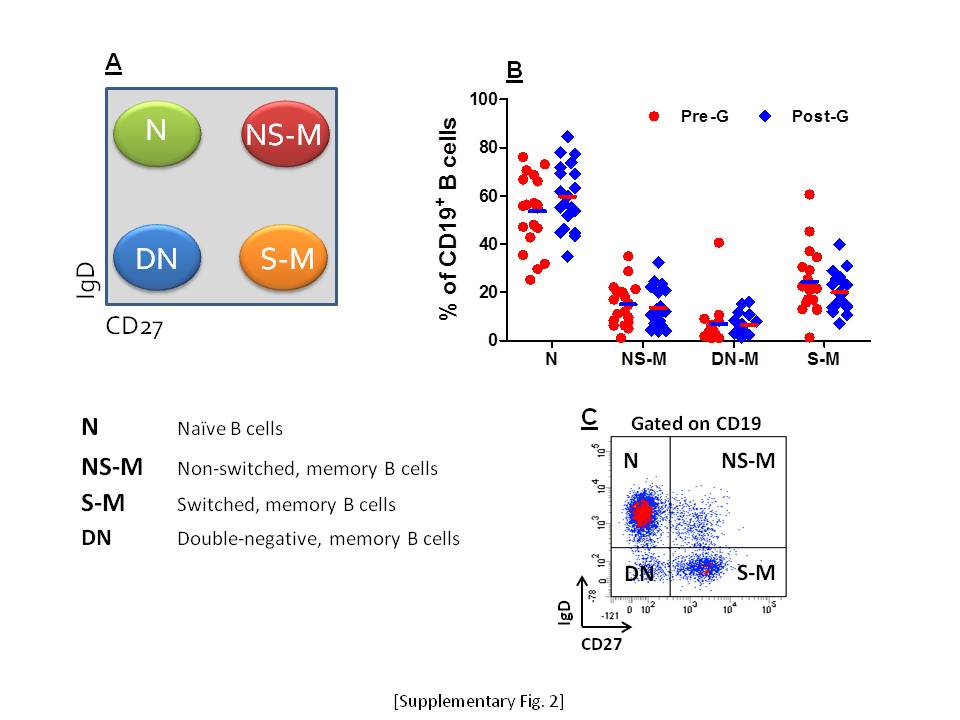

Supplement: Additional file 2: — Phenotype of circulating B-cell subsets after mobilization with G-CSF. PB from 21 randomly selected donors were analyzed for the relative frequency of naïve/memory B-cell subsets. Panel A: mAbs directed against CD19, CD27, IgM and IgD were used to discriminate naïve B cells from memory B-cell subsets [30]. Panel B: The frequency of B-cell subsets/total CD19+ B cells is shown before and after G-CSF administration. N = naïve B cells (CD19+CD27−IgD+); S-M = switched memory B cells (CD19+CD27+IgD−); NS-M = non-switched memory B cells (CD19+CD27+IgD+); DN-M = double-negative memory B cells (CD19+CD27−IgD−). Bars indicate the median value recorded in 21 independent donor samples. [file 12967_2014_240_MOESM2_ESM.jpeg]

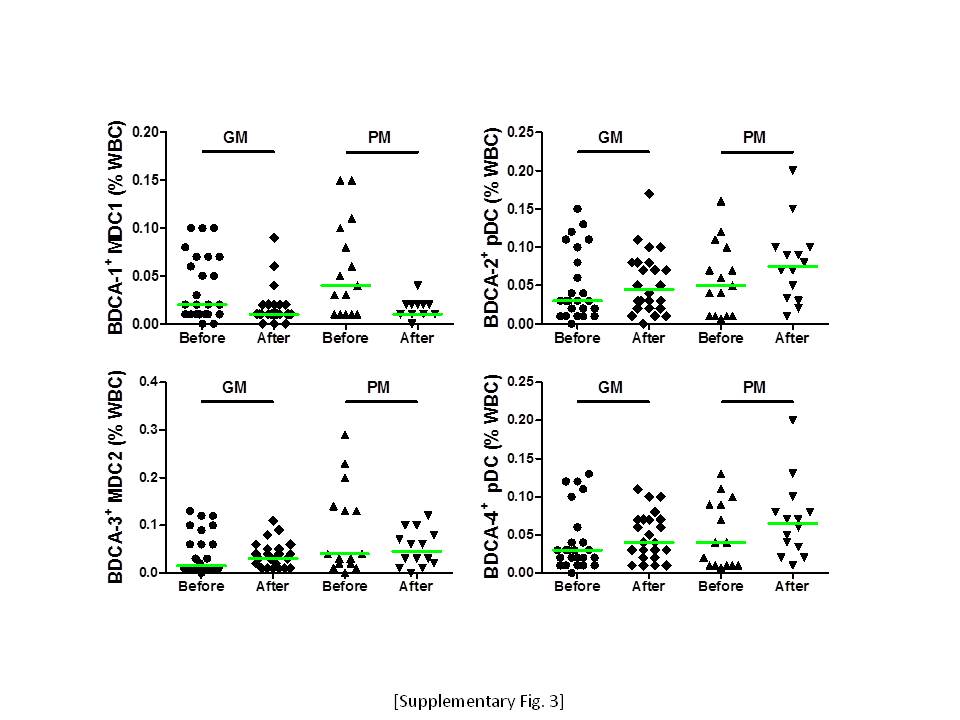

Supplement: Additional file 3: — Circulating DC subsets after mobilization with G-CSF and plerixafor (MZ). PB samples from 40 randomly selected donors (24 GMs given G-CSF alone and 16 PMs receiving G-CSF + MZ) were analyzed for the relative frequency of major DC subsets. MDC1, MDC2 and plasmacytoid DCs were identified as detailed in Materials and Methods. Bars denote the median value. Before = baseline samples; after = samples collected after HSC mobilization. [file 12967_2014_240_MOESM3_ESM.jpeg]
